# Taxon-Specific Proteins of the Pathogenic *Entamoeba* Species *E. histolytica* and *E. nuttalli*

**DOI:** 10.3389/fcimb.2021.641472

**Published:** 2021-03-19

**Authors:** Constantin König, Barbara Honecker, Ian W. Wilson, Gareth D. Weedall, Neil Hall, Thomas Roeder, Nahla Galal Metwally, Iris Bruchhaus

**Affiliations:** ^1^Bernhard Nocht Institute for Tropical Medicine, Hamburg, Germany; ^2^Institute of Infection, Veterinary & Ecological Sciences, University of Liverpool, Liverpool, United Kingdom; ^3^School of Biological and Environmental Sciences, Liverpool John Moores University, Liverpool, United Kingdom; ^4^Earlham Institute, Norwich, United Kingdom; ^5^School of Biological Sciences, University of East Anglia, Norwich, United Kingdom; ^6^Zoology, Department of Molecular Physiology, Kiel University, Kiel, Germany; ^7^Airway Research Center North (ARCN), German Center for Lung Research (DZL), Kiel, Germany; ^8^Department of Biology, University of Hamburg, Hamburg, Germany

**Keywords:** *Entamoeba*, peptidases, virulence, AIG, Ariel, BspA

## Abstract

The human protozoan parasite *Entamoeba histolytica* can live in the human intestine for months or years without generating any symptoms in the host. For unknown reasons, amoebae can suddenly destroy the intestinal mucosa and become invasive. This can lead to amoebic colitis or extraintestinal amoebiasis whereby the amoebae spread to other organs *via* the blood vessels, most commonly the liver where abscesses develop. *Entamoeba nuttalli* is the closest genetic relative of *E. histolytica* and is found in wild macaques. Another close relative is *E. dispar*, which asyptomatically infects the human intestine. Although all three species are closely related, only *E. histolytica* and *E. nuttalli* are able to penetrate their host’s intestinal epithelium. Lineage-specific genes and gene families may hold the key to understanding differences in virulence among species. Here we discuss those genes found in *E. histolytica* that have relatives in only one or neither of its sister species, with particular focus on the peptidase, AIG, Ariel, and BspA families.

## Introduction

The intestinal protozoan *Entamoeba histolytica* is an important human parasite. Recent data clearly indicate that the life-threating amoebic liver abscess (ALA) continues to be a common clinical complication of amoebiasis infection in Asian, African and Latin American countries with estimated 26700 death in 2016 (Collaborators, 2018; Shirley et al., 2019). *E. histolytica* can become invasive and cause amoebic colitis or amoebic liver abscess (ALA) formation. The life cycle of this parasite consists of infectious cysts that survive outside the host and vegetative trophozoites that proliferate in the human gut. In general, trophozoites persist asymptomatically for months or years in the human intestine. However, in 10% of cases, the trophozoites become, under as yet unknown circumstances, invasive and induce extraintestinal amoebiasis. Invasion into the intestinal mucosa can lead to induction of amoebic colitis, whereas dissemination to the liver can result in ALA formation (Blessmann et al., 2003). A related species, *E. dispar*, is microscopically indistinguishable from *E. histolytica* and occurs only as a harmless commensal in the human intestine. A key question in amoebic research is the elucidation of the mechanisms of *E. histolytica* invasion and tissue destruction. For decades, attempts have been made to identify the virulence factors of *E. histolytica* by comparative studies of both organisms at both biological and molecular level. These studies, and those that compared pathogenic and non-pathogenic *E. histolytica* isolates, have led to the identification of a number of virulence factors.

Three protein families (Gal/GalNAc lectins, cysteine peptidases and amoebapores) are of particular interest. The first step in the invasion process is to overcome the protective mucus barrier. Here, cysteine peptidases play an important role, along with a number of other molecules (Lidell et al., 2006; Thibeaux et al., 2013). Subsequently, the adhesion of amoebae to target epithelial cells *via* the galactose/N-acetyl-D-galactosamine lectin (Gal/GalNAc) (Tannich et al., 1991; Petri and Schnaar, 1995). The Gal/GalNAc lectin is a 260 kDa heterodimer consisting of a disulfide-linked 170 kDa heavy chain (Hgl) and a GPI-anchored 35 kDa light chain (Lgl) (Petri et al., 2002). After establishing contact, amoebae can secrete amoebapores. These mediate a contact-dependent lysis of the target cells (Leippe, 1997). *E. histolytica* has three amoebapores (A, B and C), which are all characterized by a pore-forming activity. They are also capable of killing gram-positive bacteria by destroying bacterial cytoplasmic membranes. Thus, the functionality of these molecules is two-fold: they confer cytolytic activity to amoebae as well as an intracellular antimicrobial effect against phagocytosed bacteria (Leippe et al., 1994). Amoebae whose amoebapore expression is inhibited have a reduced antimicrobial activity and are non-pathogenic, i.e. they are not, or are only to a small extent, able to form ALAs in hamsters (Bracha et al., 1999; Bracha et al., 2003). Important for mucus degradation, invasion, as well as for the process of tissue degradation, are the cysteine peptidases (CPs) of *E. histolytica*. In the genome of *E. histolytica* 35 genes coding for CPs of the C1 papain superfamily can be identified. However, only four CPs (EhCP-A1, -A2, -A5, -A7) can be detected at the protein level in the trophozoite stage (Tillack et al., 2007; Irmer et al., 2009). These have previously been located in lysosomal-like vesicles, and some of them were found to be membrane-associated (Jacobs et al., 1998). The importance of CPs, particularly EhCP-A5, in ALA formation is evident in infections of laboratory animals, where overexpression of CPs leads to an increase in ALA size (Tillack et al., 2006). Conversely, reduced CP activity leads to a decrease in ALA formation ability in *E. histolytica* (Li et al., 1995; Stanley et al., 1995). In addition, an increase in the expression of some *ehcp* genes during ALA formation has previously been described, while non-pathogenic amoebae can be converted to pathogenic amoebae, simply by overexpression of some of specific *ehcp* genes (Matthiesen et al., 2013).

Homologs of the Gal/GalNAc lectins, cysteine peptidases and amoebapores are also present in non-pathogenic *E. dispar* (Nickel et al., 1999; Tillack et al., 2007; Weedall et al., 2011). Nevertheless, it has been shown that the CP activity of *E. dispar* is about 10-1000 times lower than that of *E. histolytica* and that genes corresponding to *ehcp-a1* and *ehcp-a5* are absent or degenerated in *E. dispar* (Bruchhaus et al., 1996; Willhoeft et al., 1999b; Que and Reed, 2000). A lower amount of amoebapores and therefore reduced pore-forming activity was found in *E. dispar* in comparison to *E. histolytica* (Nickel et al., 1999). However, it is not yet clear whether these differences alone determine pathogenicity, whether additional genetic differences are involved, or whether pathogenicity is a result of complex interactions between various proteins.

In addition to the cysteine peptidases and the amoebapores, the AIG, BspA and Ariel families are always mentioned in connection with the virulence of *E. histolytica*. For members of the AIG family, a differential expression between pathogenic and non-pathogenic *E. histolytica* isolates could be shown (Biller et al., 2010). BspA-like molecules have also been described in *T. vaginalis* and are thought to play an important role in pathogenesis (Noel et al., 2010). The Ariel proteins are an *E. histolytica*-specific protein family that has not yet been found in any other *Entamoeba* species so far. Therefore, it is postulated that these molecules may have a role in virulence. However, the functions of the members of all three protein families are largely unclear.

Besides the characterization of individual proteins, one straightforward method for the identification of pathogenicity factors is a direct comparison of pathogenic and non-pathogenic *E. histolytica* isolates using comparative genomic, transcriptomic and proteomic approaches (Davis et al., 2006a; MacFarlane and Singh, 2006; Ehrenkaufer et al., 2007; Biller et al., 2010; Meyer et al., 2016; Nakada-Tsukui et al., 2018). Unfortunately, two isolates with very different genetic backgrounds were used in the majority of the studies cited (pathogenic isolate HM-1:IMSS and non-pathogenic isolate Rahman) (Davis et al., 2006a; MacFarlane and Singh, 2006; Davis et al., 2007; Ehrenkaufer et al., 2007). The non-pathogenic Rahman isolate has several serious functional defects (e.g. defective phagocytosis, reduced cytotoxicity, inability to grow in animals, and a truncated glycan chain of the proteophosphoglycan coating surface) (Davis et al., 2006a). The genomic differences between HM-1:IMSS and Rahman are small, however, DNA fragment duplications have been detected (Weedall et al., 2012). An alternative approach compared the transcriptomes of pathogenic and non-pathogenic clones derived from the isolate HM-1:IMSS were compared (Biller et al., 2009; Biller et al., 2010; Meyer et al., 2016). In total, approximately 90 genes are differentially expressed between the investigated non-pathogenic and pathogenic clones (Meyer et al., 2016). Based on transfectants, in which the identified genes were either overexpressed or silenced, it was possible to identify another pathogenicity factor, namely the hypothetical protein EHI_127670. When EHI_127670 is silenced in pathogenic amoebae their ability to form ALAs is reduced. On the other hand, overexpression of EHI_127670 in non-pathogenic amoebae leads to restoration of ALA formation ability (Meyer et al., 2016; Matthiesen et al., 2019). However, nothing is yet known about the function of the protein and it is not yet known with certainty that the RNA actually encodes a protein.

Using a transcriptome approach, Naiyer and colleagues were able to identify downstream regulatory motifs in very highly expressed *E. histolytica* genes that are very likely important for gene expression. These motifs were also detected in various genes encoding virulence factors, which are also highly expressed under axenic conditions. It can therefore be assumed that corresponding encoded proteins are important for optimal growth, but in addition also play a role in tissue invasion and virulence (Naiyer et al., 2019b). Comparative transcriptome analysis also makes it possible to better understand the biology of *E. histolytica* in general. With the help of this method, it is possible to identify molecules that play a role in phagocytosis, the stress response, the enzyme station and the excystation, among others [for review (Naiyer et al., 2019a)].

Besides *E. histolytica* and *E. dispar*, the genus *Entamoeba* includes many other species, some of which colonize the human intestine (*E. moshkovskii, E. bangladeshi, E. polecki, E. coli*, and *E. hartmanni*). However, in humans a severe course of disease with extraintestinal abscesses has so far only been described for *E. histolytica*. Pathogenic amoebae have also been described in reptiles (*E. invadens*) and macaques (*E. nuttalli*). *E. nuttalli* is the species most closely related to *E. histolytica*. Different species of wild macaques, as well as other non-human primates kept in captivity, have been identified as hosts for *E. nuttalli* (Tachibana et al., 2007; Tachibana et al., 2009; Levecke et al., 2010; Wei et al., 2018; Tanaka et al., 2019). In addition to the genomes of *E. histolytica* and *E. dispar*, the genome of *E. nuttalli* was recently sequenced and all genomes are available on AmoebaDB (Tanaka et al., 2019). The ability to compare the genomes of *E. histolytica*, *E. dispar* and *E. nuttalli* now opens up the possibility of identifying additional molecules responsible for the development of extraintestinal amoebiasis in human or non-human primates.

## Identification of Homologous Proteins Unique to Pathogenic *E. histolytica* and *E. nuttalli*

**Figure 1:**
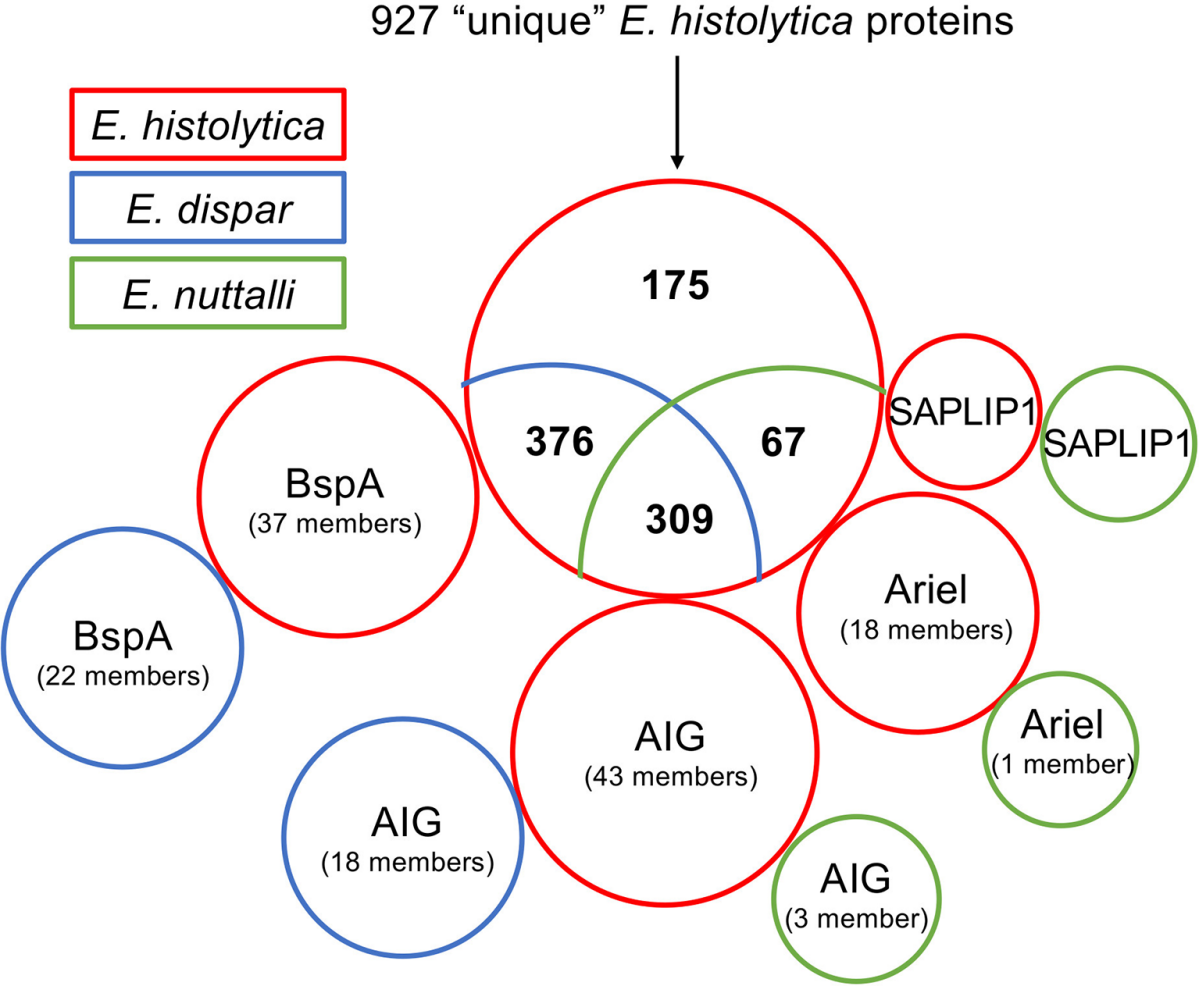
Comparative blastp analysis of 927 proteins identified as unique for *E. histolytica* (Wilson et al., 2019). Shown is the comparison of *E. histolytica* with *E. nuttalli* and *E. dispar* as a venn diagram. The three protein families Ariel, AIG and BspA as well SAPLIP1 are shown separately. The proteins found in *E. histolytica* are boxed in red, those of *E. dispar* in blue and those of *E. nuttalli* in green.

Recently, using an OrthoMCL approach, Wilson and colleagues studied the genetic diversity and gene family expansions for *E. histolytica*, *E.dispar*, *E. moshkovskii* and *E. invadens*. They identified 984 genes that were unique to *E. histolytica* (**Table S8**) (Wilson et al., 2019). Here, this list was used as a basis for a BlastP approach to analyze whether homologous proteins are found in *E. nuttalli*. Additionally, *E. dispar* was again included in this analysis. Pseudogenes were not included in the analysis and a sequence identity of ≥ 50% was assumed to be a functionally homologous protein. In a BlastP analysis of all 927 translatable “unique” *E. histolytica* genes, 309 of them had homologs in *E. dispar* and *E. nuttalli* with an identity of ≥ 50% (**Figure 1**, **Table S7**) (Wilson et al., 2019). Another 376 “unique” *E. histolytica* genes encode for proteins with homologs in *E. dispar* but not in *E. nuttalli* (**Figure 1**, **Table S3**). A further 67 putative proteins share ≥ 50% identity only with *E. nuttalli* proteins (**Figure 1**, **Table S6**). Only 175 genes remain, whose encoded proteins do not show homology with proteins of *E. dispar* or *E. nuttalli* (**Figure 1**, **Table S1**). That many of the proteins encoded by the “unique” *E. histolytica* genes were also found in *E. dispar* was very surprising. However, looking at the genomic organization on AmoebaDB, one finds that many of the “unique” *E. histolytica* genes are located at the edge of contigs and therefore do not contain the complete gene sequence (Wilson et al., 2019).

## Proteins That Could Only Be Detected in *E. histolytica*

Almost all 175 proteins found to be unique for *E. histolytica* are hypothetical proteins (**Table S1**). However, 18 genes encoding the asparagine-rich *E. histolytica* antigens (Ariel) could be identified (including two genes with an incomplete sequence) (**Figure 1**, **Table S2**) (Mai et al., 2000; Wilson et al., 2019). The fact that Ariel proteins are encoded by an *E. histolytica*-specific multicopy gene family was described by Willhoeft and colleagues 20 years ago (Willhoeft et al., 1999a). No homologous proteins can be detected in *E. dispar* or *E. invadens* (Wilson et al., 2019). However, one homologous protein (ENU1_012640) is found in *E. nuttalli*, with 92% identity to EHI_005260/EHI_188600. Except for the Ariel proteins EHI_057430 and EHI_172730, which did not contain a signal peptide, and EHI_131360 and EHI_185110 which did not contain a transmembrane domain, an N-terminal signal peptide and a C-terminal transmembrane domain can be predicted for all other Ariel proteins. Therefore, a surface localization can be postulated, however it is unknown whether the protein is a deterministic factor in the virulence of *E. histolytica*.

**Figure 2:**
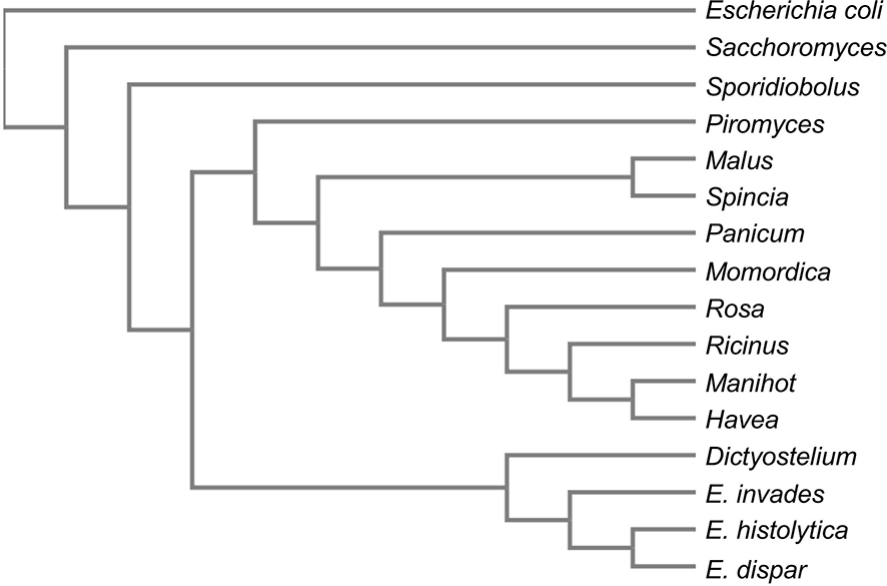
Phylogram of aldose reductases. The aldose reductases sequences of *E. histolytica*, *E. dispar*, *E. nuttalli* were compared for homologous sequences using BlastP. The best scoring 10 proteins as well as three aldose reductase sequences used as outgroups were used to generate a phylogram using the online tool Clustal Omega (Sievers et al., 2011). Sequences used: *E. histolytica* aldose reductase (EHI_029620/EHI_039190), *E. invadens* aldo_ket_red domain-containing protein (EIN_497000), *E. dispar* NADPH-dependent alpha-keto amide reductase (EDI_260680), *Piromyces finnis*_aldehyde reductase_(ORX59359), *Ricinus communis*_NADPH-dependent aldo-keto reductase, chloroplastic_(XP_002529872), *Manihot esculenta*_NADPH-dependent aldo-keto reductase, chloroplastic-like_(XP_021619253), *Hevea brasiliensis*_NADPH-dependent aldo-keto reductase, chloroplastic-like (XP_021670007), *Momordica charantia*_NADPH-dependent aldo-keto reductase, chloroplastic-like_(XP_022143954), *Dictyostelium discoideum*_aldehyde reductase_(XP_628918), *Malus domestica* NADPH-dependent aldo-keto reductase, chloroplastic-like (XP_008369945), *Spinacia oleracea*_NADPH-dependent aldo-keto reductase, chloroplastic-like (XP_021845528), *Panicum hallii* aldo-keto reductase family 4 member C10-like isoform X2 (XP_025809823), *Rosa chinensis*_NADPH-dependent aldo-keto reductase, chloroplastic-like (XP_024188589), *Escherichia coli* aldo-keto reductase (E0IVZ7), *Saccharomyces cerevisiae* NADPH-dependent aldose reductase (P38715), *Sporidiobolus salmonicolor* aldehyde reductase (P27800).

Two further genes unique to *E. histolytica* unique genes (EHI_107560 and EHI_157010) encode for proteins with approximately 55% sequence identity to aldo-keto reductases of plant chloroplasts and are annotated as alcohol dehydrogenases. However, both genes are located at the edges of contigs and are thus only partially represented in the genome assembly. EHI_107560 (187 aa) is located at the edge of contig DS571548, while EHI_157010 (158 aa) is located at the edge of contig DS571869 (AmoebaDB, release 48 beta, 20 Aug 2020). BlastP analysis of these proteins revealed two genes (EHI_029620, EHI_039190) in the genome of *E. histolytica* encoding the same protein sequence of 305 amino acids, termed aldolase reductase. If these proteins are used as a template for a search in the *Entamoeba* genomes, a protein with a sequence identity of 55% was also found in pathogenic *E. invadens* (EIN_497000), along with one with 73% sequence identity in *E. dispar* (EDI_260680). A phylogenetic analysis using the online tool Clustal Omega with the respective protein sequences as input (Sievers et al., 2011), showed the closest relationship to *Dictyostelium discoideum* and plant chloroplastic-like aldo-keto reductases (**Figure 2**). In various Kinetoplastidae such as *Trypanosoma* and *Leishmania*, genes encoding chloroplast-like proteins were identified. It is assumed that the organelles were taken up by endosymbiosis before divergence. Later, the organelles were lost, but several genes from these organelles were integrated into the genome (Hannaert et al., 2003). Horizontal gene transfer has also been described for *Entamoeba*. However, almost all identified genes could be traced back to bacteria (mainly Bacterioidetes) so far (Romero et al., 2016). Comparison of the amino acid sequences revealed that *E. dispar* EDI_260680, contains a deletion of about 50 aa. Comparison of the sequence with those of plant aldo-keto reductases shows that this area contains amino acids that are important for the enzymes’ activity (Sengupta et al., 2015). In plants aldo-keto reductases play a role in stress response and detoxification of toxic aldehydes among other things (Sengupta et al., 2015). Since nothing is known about the function of the protein in *E. histolytica*, no statement can be made as to whether the deletion present in *E. dispar* has an influence on its activity.

## Proteins Occurring in *E. histolytica* and *E. dispar*, but Not in *E. nuttalli*

In the gene set of *E. histolytica* “unique” genes underlying this work, 37 genes encoding members of the BspA family (leucine-rich repeat protein) were identified (Wilson et al., 2019). BlastP analysis of the proteins encoded by *bspa* showed that for all but two (EHI_098720/EHI_173850: 49% identity), homologous proteins (in total 22 members) with an identity of ≥ 50% could be detected in *E. dispar* (**Figure 1**, **Tables S3** and **S4**). However, no homologous proteins could be found in *E. nuttalli*. The length of *bspa*-encoded proteins listed here ranges from 101 to 447 aa. It is striking that almost all *bspa*s are found on very short contigs and then often on the edge of the contigs. Therefore, it is not clear whether all of these are really full-length proteins since, with a few exceptions, the BspA proteins characterized so far have an average length of about 550 amino acids (Davis et al., 2006b). For some members of the BspA family a surface localization was described, although neither signal sequences nor transmembrane domains were detected (Davis et al., 2006b, Silvestre et al., 2015). However, the phenomenon of a surface localization without the detection of protein domains that would allow membrane anchoring has been described for a number of other proteins (Biller et al., 2014). It has been shown that BspA-like proteins are involved in adhesion to extracellular membranes, epithelial cell invasion and fibronectin and fibrinogen binding (Mengaud et al., 1996; Sharma et al., 1998; Inagaki et al., 2006). A BspA-like gene family could also be identified in *Trichomonas vaginalis* (Hirt et al., 2002; Noel et al., 2010). The BspA proteins of *T. vaginalis* are believed to play various and important roles in the pathobiology of this parasite by contributing to invasion and long-term infections of the urogenital tract (Noel et al., 2010). However, the exact functions of the proteins in *T. vaginalis* as well as in *E. histolytica* and *E. dispar* are not yet deciphered. As these are large gene families, it can be assumed that they play an important role in the life cycle of the amoebae. It is therefore all the more surprising that homologous proteins are not detectable in the closest relative of *E. histolytica*, *E. nuttalli*.

Wilson and colleagues found 17 members of the AIG family that were “unique” to *E. histolytica* (Wilson et al., 2019). However, by BlastP analysis 8 AIG proteins were identified in *E. dispar*, showing 49 – 77% homology to the 17 different *E. histolytica* AIG1 proteins. Eight of the *E. histolytica* AIGs are most similar (53 – 63% identity) to *E. dispar* EDI_185310. For only one AIG1, EHI_126560, homologs were identified in both *E. dispar* (EDI_274460, 77% identity) and *E. nuttalli* (ENU1_158210, 97% identity)(**Tables S3**, **S5**). In addition to the 17 genes, 26 more genes encoding AIG proteins can be identified in the *E. histolytica* genome. For all these proteins, homologs with a sequence identity between 53 and 83% can be detected in *E. dispar*. However, seven *E. histolytica* AIGs each show the highest homology to only two *E. dispar* AIGs (EDI_036000 and EDI_243490). Furthermore, two more AIGs, ENU1_161270 and ENU1_207600, are found in *E. nuttalli*, which are homologous to AIGs from *E. histolytica* (EHI_180390, 95% identity, and EHI_191790, 98% identity, respectively) (**Figure 1**, **Table S5**).

AIGs belong to the GTPases, were originally isolated from *Arabidopsis thaliana* and are thought to confer resistance to bacterial infections. It is also believed that AIG proteins are involved in the development of *A. thaliana* and its response to environmental stimuli (Reuber and Ausubel, 1996). Orthologous proteins are also found in mammals and play a role in B-cell and T-cell development *via* interaction with proteins of the Bcl2 family (Nitta et al., 2006; Nitta and Takahama, 2007). However, very little is known about the function of AIG proteins in *E. histolytica*. In a comparative genome analysis of *E. histolytica* isolated from a patient presenting with diarrhea and an asymptomatic patient, it was shown that one *aig* gene from a tandem array of three *aig* genes was deleted by homologous recombination in the isolate from the asymptomatic patient. Overexpression of this *aig* (EHI_176590) resulted in increased formation of cell surface protrusions and increased adhesion to human erythrocytes. Furthermore, the gene EHI_176590 was detected in approximately 60% of stool samples from symptomatic patients, but only in 15% of stool samples from asymptomatic individuals infected with *E. histolytica*. It is therefore postulated that the AIG protein plays a central role in the virulence of *E. histolytica* by regulating host cell adhesion (Nakada-Tsukui et al., 2018). In addition, a quantitative real-time PCR approach showed that 18 of 34 investigated *aig* genes are increasingly expressed in pathogenic compared to non-pathogenic HM-1:IMSS cell lines (Biller et al., 2010). But as with the Ariel family and the BspA family, the function of the AIG family is not yet clear.

## Proteins Occurring in *E. histolytica* and *E. nuttalli*, but Not in *E. dispar*

In the set of “unique” *E. histolytica* genes on which this work is based, 67 had homologs in *E. nuttalli* but not in *E. dispar* (**Figure 1**, **Table S6**) (Wilson et al., 2019). As already described for the other comparisons, these are mostly hypothetical proteins. However, the list also contains the non-pathogenic pore-forming peptide EHI_169350. EHI_169350 (entbd24tf or SAPLIP1) belongs to a family of 15 saposin-like proteins (SAPLIPs) which were first identified by Bruhn and Leippe (Bruhn and Leippe, 2001; Winkelmann et al., 2006). Like amoebapore A, B and C the SAPLIPs are characterized by a conserved sequence motif consisting of six cysteine residues. Interestingly, of the entire SAPLIP family, only EHI_169350 can be clearly assigned to the amoebapore subfamily, with the largest sequence identity of about 65% shared with amoebapore A (Bruhn and Leippe, 2001). Only two members of the SAPLIP family including EHI_169350 possess a typical signal peptide and only EHI_169350 resembles amoebapores in its net charge and dispersed charge distribution, with the greatest similarity being to amoebapore C. However, EHI_169350 lacks the typical C-terminal histidine residue of amoebapores, which is essential for oligomerization during channel formation (Andra and Leippe, 1994). EHI_169350 has an asparagine residue at position 90 instead. It can therefore be assumed that EHI_169350 has no pore-forming activity (Bruhn and Leippe, 2001). No statement can therefore be made about the function of EHI_169350 especially concerning its role in pathogenicity.

## The Peptidases of E. histolytica, E. dispar and E. nuttalli

**Figure 3:**
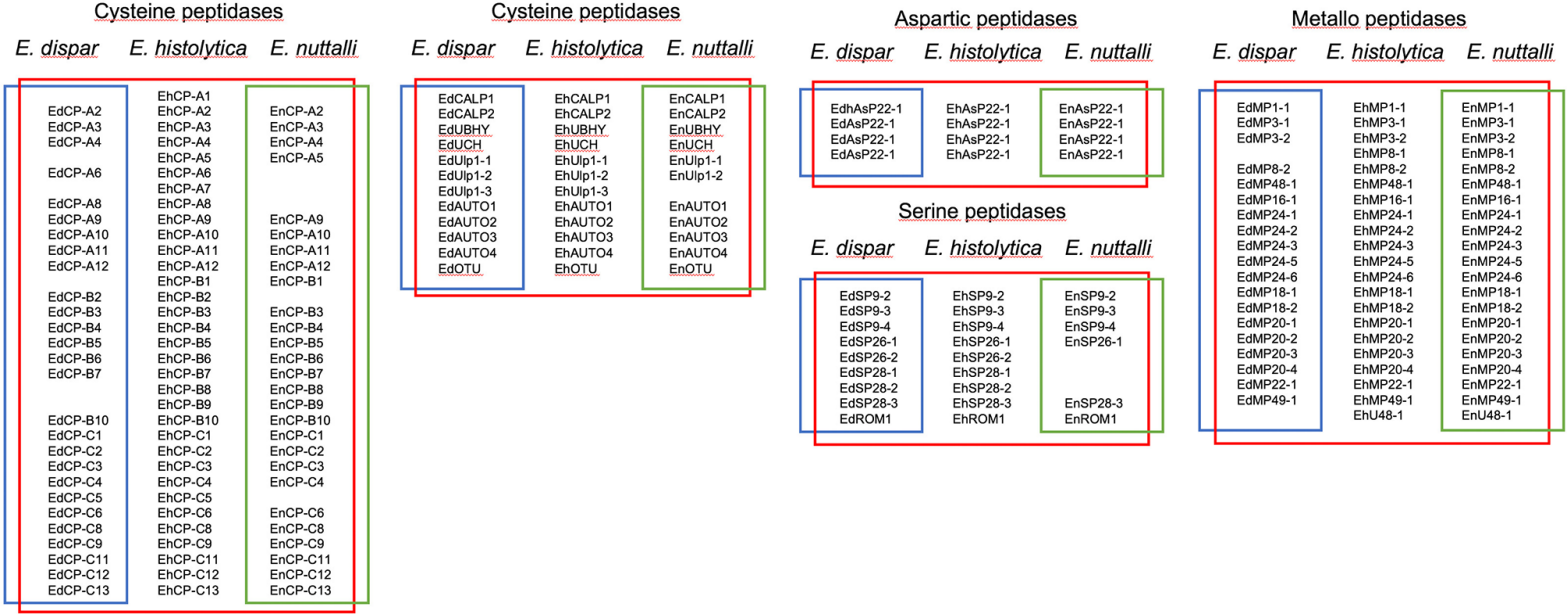
Cysteine, asparagine, serine and metallopeptidases of *E. histolytica*, *E. dispar* and *E. nuttalli* (for more details see **Table S9**).

As described above, CPs play an important role in the destruction and invasion of human tissue. This has been demonstrated in a variety of *in vitro* and *in vivo* studies (Bruchhhaus and J, 2015; Gilmartin et al., 2020). A new *in silico* analysis of the genome of *E. histolytica* (AmoebaDB, release 48 beta, 27 Aug 2020) revealed a total of 33 genes encoding CPs of clan CA, C1 (papain-like) family (**Table S9**). Twelve of the CPs could be assigned to the CP-A family, ten to the CP-B family and eleven to the CP-C family. In *E. dispar* there are only nine members of the CP-A family. Genes coding for EdCP-A1, -A5, -A7 are not present or are present as pseudogenes. The CP-B family consists of seven members; here the genes coding for EdCP-B1, -B8 and -B9 are missing. The EdCP-C family has eleven members, like the EhCP-C family. In *E. nuttalli* there are no homologous proteins to EhCP-A1, -A6, -A7, and -A8; thus the EnCP-A family comprises eight members. The EnCP-B family consists of nine members (EnCP-B2 is missing here), and EnCP-C5 is missing in the CP-C family, so there are ten members in total (**Figure 3**, **Table S9**).

Only for four of the CP encoding genes (*ehcp-a1, -a2, -a5* and -*a7*) could it be shown that they are highly expressed in *E. histolytica* under the standard axenic culture conditions (Bruchhaus et al., 2003; Clark et al., 2007; Tillack et al., 2007). Interestingly, the ability to disrupt a cell monolayer (cytopathic activity) was dramatically increased for amoebae overexpressing *ehcp-a5*, whereas it showed only a moderate increase in transfectants overexpressing *ehcp-a1* or *ehcp-a2*. Furthermore, the overexpression of *ehcp-a1* and *ehcp-a2* in *E. histolytica* trophozoites did not increase ALA formation in gerbils, whereas overexpression of *ehcp-a5* resulted in significantly larger ALAs compared to controls. If the *ehcp5* is overexpressed in the HM-1:IMSS derived G3 isolate, in which the *amoebapore a g*ene is silenced and therefore has only low virulence, this is sufficient to compensate for the reduction in virulence (Hellberg et al., 2001; Tillack et al., 2006). In previous studies EhCP-A5 was also believed to play an important role in intestinal invasion. EhCP-A5 has been shown to interact directly with the integrins on the surface of human colon epithelial cells and induce the secretion of pro-inflammatory cytokines (Hou et al., 2010). Thus, amoebae silenced for *ehcp-a5* expression do not trigger an inflammatory response of the host and do not induce the collagen remodeling required for invasion. Further investigations showed that CP-A5 can convert the pro-matrix metalloproteinase (MMP)-3 into its active form, which in turn activates pro-MMP-1 (Bansal et al., 2009; Thibeaux et al., 2012; Thibeaux et al., 2014). The observation that CP-A5 occurs in the two pathogenic amoeba species *E. histolytica* and *E. nutalli*, but not in the non-pathogenic species *E. dispar*, supports the significance of this peptidase as an important pathogenicity factor (**Figure 3**, **Table S9**). However, a CP-A5 homologue is not detectable in the genome of the reptile pathogen *E. invadens*.

In the genome of *E. histolytica* another twelve genes encoding CPs can be identified and assigned to five different families (C2, C19, C48, C54, C65; all clan CA) (**Figure 3**, **Table S9**). Except for the Ulp1 protease Ulp1-3 (C48 family) in *E. nuttalli*, homologs for all CPs in *E. dispar* and *E. nuttalli* could be found.

Homologs for the four members of the aspartic peptidase family (clan AD, family A22, A) of *E. histolytica* are found in both *E. dispar* and *E. nuttalli*. However, nothing is known about the function of these peptidases (**Figure 3**, **Table S9**).

Nine serine peptidases can be detected, which can be assigned to four families (clan SC, family S9, C; Clan SF, family S26, B; Clan SC, S28; clan ST, family S54) (**Figure 3**, **Table S9**). For the serine peptidases EhSP26-2, EhSP28-1 and EhSP28-2 no homologs can be detected in *E. nutalli*. Functional analysis has only been performed for the S28 and rhomboid proteases. Both are found associated with the amoeba membrane and the rhomboid protease probably plays an important role in mobility and adhesion of amoebae to the host tissue (Barrios-Ceballos et al., 2005; Baxt et al., 2008; Rastew et al., 2015; Welter et al., 2020).

A total of 21 metallopeptidases belonging to eleven different families were identified in the genome of *E. histolytica* (**Figure 3**, **Table S9**). Only for 2 of them no homologous proteins can be identified in *E. dispar*. These are MP8-1 (cell surface protease gp63) and U48-1, a CAAX prenyl protease. In *E. nuttalli* the two peptidases can be detected, just like in *E. histolytica*. For EhMP8-1, Teixeira and colleagues showed that it is a functional metallopeptidase localized on the surface of E. histolytica trophozoites. By silencing *ehmp8-1* expression, the adherence of trophozoites to cells was increased, while the surface staining of several antigens, including the Gal/GalNAc lectin, remained unchanged. Amoebae which were silenced for *ehmp8-1* expression also showed decreased cytopathic activity and reduced mobility, but phagocytic activity was increased (Teixeira et al., 2012). In contrast to the EhMP8-1 there is unfortunately very little information about the EhMP8-2. The *ehmp8-2* gene is approximately 150 times more highly expressed in non-pathogenic amoebae than in pathogenic amoebae. Furthermore, it was shown that overexpression of *ehmp8-2* in pathogenic amoebae significantly reduced ALA formation in the mouse model. Thus, the presence of EhMP8-2 leads to a non-pathogenic phenotype of the amoebae (Meyer et al., 2016). Another peptidase, which is found only in *E. histolytica* and *E. nuttalli*, but not in *E. dispar*, is a CAAX prenyl protease of unknown function in the U48 family. The question arises for EhMP8-2 as well as for EhU48-1 whether the absence of these molecules has an influence on the virulence of amoebae.

## Conclusion

Unfortunately, the majority of proteins found exclusively in pathogenic *E. histolytica*, or in *E. histolytica* and *E. nuttalli* but not non-pathogenic *E. dispar*, lack functional annotations. With Ariel, BspA and AIG, *E. histolytica* has three large protein families whose members are repeatedly discussed as virulence factors. The Ariel family, consisting of 18 members, is almost exclusively found in *E. histolytica*. Only one member was detected in *E. nuttalli*. In contrast, proteins of the BspA family are found in *E. histolytica* and *E. dispar*, and members of the AIG family in all three organisms. However, it is noticeable that *E. histolytica* contributes the most members to each of these protein families. One reason for this observation could be that, in analyses like these, incomplete or fragmented genome assemblies may contain gaps and genes may be missing in the annotated gene set rather because of this than because of the actual absence of these genes. For example, the lysine and glutamic acid-rich protein KERP1 is a virulence factor that is active in the development of amoebic liver abscesses (Seigneur et al., 2005; Santi-Rocca et al., 2008). Originally, it was assumed to be absent in the genome of *E. dispar*, but in fact it is present but only partially represented in the genome assembly (Weedall, 2020). Such a situation is probably a factor in defining genes as lineage-specific. It is probably also a wide-ranging confounding factor for *Entamoeba* genomes, which are particularly difficult to assemble for a number of reasons (Weedall and Hall, 2011), and is likely to have a greater impact on genome assemblies with low coverage such as *E. dispar*. Therefore, the correctness of the genome assembly should first be verified before further investigations on the influence of the *E. histolytica*-unique proteins on virulence are performed.

## Author Contributions

All authors (JK, BH, IW, GDW, NH, TR, NM, and IB) wrote, edited, and reviewed the drafts of the manuscript, and agree to be accountable for the content of the work. All authors contributed to the article and approved the submitted version.

## Funding

This work was supported by the Deutsche Forschungsgemeinschaft (BR1744/17-1), Joachim Herz Stiftung (Joachim Herz Graduate School (BH)) und Jürgen Manchot Stiftung (CK).

## Conflict of Interest

The authors declare that the research was conducted in the absence of any commercial or financial relationships that could be construed as a potential conflict of interest.
