# Supplementary material for: Taxon-Specific Proteins of the Pathogenic Entamoeba Species E. histolytica and E. nuttalli
Source: Front Cell Infect Microbiol. 2021 Mar 19;11:641472. doi: 10.3389/fcimb.2021.641472 (PMC8017271; doi:10.3389/fcimb.2021.641472)
Supplement: Supplementary file 1 [file DataSheet_1.zip › Table S9.docx]

Table S9 Peptidases of *E. histolytica*, *E. dispar* and *E. nuttalli*

| Clan, Family | Name | Accession number | Length (aa) | Identity (%) | Name, NCBI |
| --- | --- | --- | --- | --- | --- |
| **Cysteine peptidases** | |  |  |  |  |
| CA, C1, A | EhCP-A1 | EHI_074180 | 315 | / | cysteine protease 1 |
|  |  | **EDI_171580** | 315 | 82 | cysteine proteinase 3 |
|  |  | **ENU1_055850** | 315 | 87 | cysteine proteinase 2 |
|  | EhCP-A2 | EHI_033710 | 315 | / | cysteine proteinase 2 |
|  | EdCP-A2 | EDI_171580 | 315 | 93 | cysteine proteinase 3 |
|  | EnCP-A2 | ENU1_055850 | 315 | 96 | cysteine proteinase 2 |
|  | EhCP-A3 | EHI_159610 | 308 | / | cysteine protease |
|  | EdCP-A3 | EDI_039310 | 308 | 96 | cysteine proteinase ACP1 |
|  | EnCP-A3 | ENU1_171930 | 308 | 98 | cysteine protease |
|  | EhCP-A4 | EHI_050570 | 311 | / | cysteine proteinase |
|  | EdCP-A4 | EDI_156850 | 311 | 92 | cysteine proteinase 3 |
|  | EnCP-A4 | ENU1_143540 | 311 | 95 | cysteine proteinase |
|  | EhCP-A5 | EHI_168240 | 318 | / | cysteine proteinase |
|  |  | **EDI_–** | – | – |  |
|  | EnCP-A5 | ENU1_172220 | 318 | 97 | cysteine proteinase |
|  | EhCP-A6 | EHI_151440 | 320 | / | cysteine proteinase |
|  | EdCP-A6 | EDI_113090 | 320 | 91 | cysteine proteinase 3 |
|  |  | **ENU1_143540** | 322 | 63 | cysteine proteinase |
|  | EhCP-A7 | EHI_039610 | 315 | / | cysteine proteinase |
|  | EhCP-A7 | EHI_010850 | 315 | 99 | cysteine proteinase, |
|  |  | **EDI_171580** | 315 | 72 | cysteine proteinase 3 |
|  |  | **ENU1_055850** | 316 | 73 | cysteine proteinase 2 |
|  | EhCP-A8 | EHI_151400 | 317 | / | cysteine proteinase |
|  | EdCP-A8 | EDI_113060 | 320 | 82 | cysteine proteinase ACP1 |
|  |  | **ENU1_143540** | 311 | 56 | cysteine proteinase |
|  | EhCP-A9 | EHI_096740 | 297 | / | cysteine proteinase |
|  | EdCP-A9 | EDI_092720 | 327 | 97 | cysteine proteinase 2 |
|  | EnCP-A9 | ENU1_055550 | 327 | 98 | cysteine proteinase |
|  | EhCP-A10 | EHI_062480 | 420 | / | cysteine protease |
|  | EdCP-A10 | EDI_252380 | 419 | 98 | cathepsin L |
|  | EnCP-A10 | ENU1_117300 | 420 | 99 | cysteine protease |
|  | EhCP-A11 | EHI_197490 | 324 | / | cysteine protease |
|  | EdCP-A11 | EDI_002840 | 324 | 90 | cysteine proteinase 2 |
|  | EnCP-A11 | ENU1_000900 | 324 | 97 | cysteine protease |
|  | EhCP-A12 | EHI_180170 | 317 | / | cysteine protease |
|  | EdCP-A12 | EDI_243160 | 317 | 90 | cathepsin H |
|  | EnCP-A12 | ENU1_191380 | 317 | 98 | cysteine protease |
|  | EhCP-B1 | EHI_117650 | 426 | / | cysteine protease |
|  | EdCP-B1 | **EDI_029570** | 431 | 81 | Gut-specific cysteine proteinase recursor |
|  | EnCP-B1 | ENU1_037270 | 426 | 93 | cysteine protease |
|  | EhCP-B2 | EHI_179600 | 431 | / | cysteine protease |
|  | EhCP-B2 | EHI_084060 | 431 | 100 | cysteine protease |
|  | EhCP-B2 | EDI_029570 | 431 | 84 | Gut-specific cysteine proteinase precursor |
|  |  | **ENU1_037270** | 426 | 82 | cysteine protease |
|  | EhCP-B3 | EHI_140220 | 474 | / | cysteine protease |
|  | EdCP-B3 | EDI_043410 | 470 | 90 | cathepsin Q |
|  | EnCP-B3 | ENU1_048070 | 474 | 100 | cysteine protease |
|  | EhCP-B4 | EHI_030720 | 444 | / | cysteine protease |
|  | EdCP-B4 | EDI_082600 | 443 | 85 | cysteine protease |
|  | EnCP-B4 | ENU1_054620 | 443 | 95 | cysteine protease |
|  | EhCP-B5 | EHI_200690 | 374 | / | cysteine protease |
|  | EdCP-B5 | EDI_315330 | 435 | 91 | cathepsin W |
|  | EnCP-B5 | ENU1_025800 | 374 | 98 | cysteine protease |
|  | EhCP-B6 | EHI_126170 | 316 | / | cysteine protease |
|  | EdCP-B6 | EDI_012640 | 300 | 87 | cysteine proteinase ACP1 |
|  | EnCP-B6 | ENU1_044600 | 316 | 96 | cysteine protease |
|  | EhCP-B7 | EHI_091450 | 650 | / | cysteine protease |
|  | EdCP-B7 | EDI_217900 | 650 | 90 | cysteine proteinase 2 |
|  | EnCP-B7 | ENU1_017600 | 650 | 99 | cysteine protease |
|  | EhCP-B8 | EHI_097900 | 473 | / | cysteine protease |
|  |  | **EDI_057770** | 451 | 50 | cysteine protease |
|  | EnCP-B8 | ENU1_002660 | 473 | 95 | cysteine protease |
|  | EhCP-B9 | EHI_181230 | 446 | / | cysteine protease |
|  |  | **EDI_057770** | 451 | 50 | cysteine protease |
|  | EnCP-B9 | ENU1_185840 | 446 | 98 | cysteine protease |
|  | EhCP-B10 | EHI_180650 | 339^a^ | / | cysteine protease |
|  | EdCP-B10 | EDI_057770 | 451 | 89 | cysteine protease |
|  | EnCP-B10 | ENU1_003030 | 234^a^ | 96 | cysteine protease |
|  | EhCP-C1 | EHI_006920 | 571 | / | papain family cysteine protease domain containing protein |
|  | EdCP-C1 | EDI_276390 | 373 | 97 | hypothetical protein |
|  | EnCP-C1 | ENU1_001730 | 571 | 99 | papain family cysteine protease |
|  | EhCP-C2 | EHI_050800 | 567 | / | hypothetical protein |
|  | EdCP-C2 | EDI_203900 | 567 | 95 | hypothetical protein |
|  | EnCP-C2 | ENU1_023620 | 567 | 99 | papain family cysteine protease |
|  | EhCP-C3 | EHI_138460 | 572 | / | papain family cysteine protease domain containing protein |
|  | EdCP-C3 | EDI_064860 | 562 | 99 | hypothetical protein |
|  | EnCP-C3 | ENU1_023620 | 567 | 99 | papain family cysteine protease |
|  | EhCP-C4 | EHI_152220 | 502 | / | hypothetical protein |
|  | EdCP-C4 | EDI_168350 | 502 | 97 | hypothetical protein |
|  | EnCP-C4 | ENU1_079020 | 572 | 99 | papain family cysteine protease |
|  | EhCP-C5 | EHI_010340 | 554 | / | hypothetical protein |
|  | EdCP-C5 | EDI_255230 | 554 | 95 | hypothetical protein |
|  |  | **ENU1_079410** | 572 | 46 | hypothetical protein |
|  | EhCP-C6 | EHI_127030 | 503 | / | hypothetical protein |
|  | EdCP-C6 | EDI_190570 | 550 | 97 | hypothetical protein |
|  | EnCP-C6 | ENU1_057430 | 550 | 99 | hypothetical protein |
|  | EhCP-C8 | EHI_182770 | 627 | / | hypothetical protein |
|  | EdCP-C8 | EDI_251610 | 627 | 94 | hypothetical protein |
|  | EnCP-C8 | ENU1_024020 | 627 | 99 | papain family cysteine protease domain containing protein |
|  | EhCP-C9 | EHI_104450 | 538 | / | hypothetical protein |
|  | EdCP-C9 | EDI_077850 | 538 | 96 | hypothetical protein |
|  | EnCP-C9 | ENU1_116940 | 538 | 99 | hypothetical protein |
|  | EhCP-C11 | EHI_083110 | 526 | / | hypothetical protein |
|  | EdCP-C11 | EDI_077840 | 523 | 95 | hypothetical protein |
|  | EnCP-C11 | ENU1_208880 | 526 | 99 | hypothetical protein |
|  | EhCP-C12 | EHI_197820 | 488 | / | hypothetical protein |
|  | EdCP-C12 | EDI_241600 | 488 | 94 | hypothetical protein |
|  | EnCP-C12 | ENU1_061120 | 488 | 99 | hypothetical protein |
|  | EhCP-C13 | EHI_093970 | 591 | / | hypothetical protein |
|  | EdCP-C13 | EDI_146070 | 595 | 97 | hypothetical protein |
|  | EnCP-C13 | ENU1_206770 | 591 | 99 | calpain large subunit domain III containing protein |
| CA, C2 | EhCALP1 | EHI_146330 | 591 | / | calpain large subunit domain III containing protein |
|  | EdCALP1 | EDI_313350 | 591 | 97 | hypothetical protein |
|  | EnCALP1 | ENU1_206770 | 591 | 99 | calpain large subunit domain III containing protein |
|  | EhCALP2 | EHI_045290 | 473 | / | calpain family cysteine protease |
|  | EdCALP2 | EDI_245360 | 442^a^ | 97 | calpain |
|  | EnCALP2 | ENU1_047310 | 473 | 98 | calpain family cysteine protease |
| CA, C19 | EhUBHY | EHI_152110 | 444 | / | ubiquitin carboxyl-terminal hydrolase domain containing protein |
|  | EdUBHY | EDI_038430 | 427 | 89 | pre-mRNA-splicing factor SAD1 |
|  | EnUBHY | ENU1_091700 | 375 | 97 | ubiquitin carboxyl-terminal hydrolase domain containing protein |
|  | EhUCH | EHI_049540 | 386 | / | ubiquitin carboxyl-terminal hydrolase domain containing protein |
|  | EdUCH | EDI_336480 | 379 | 92 | ubiquitin carboxyl-terminal hydrolase |
|  | EnUCH | ENU1_000510 | 336 | 98 | ubiquitin carboxyl-terminal hydrolase domain containing protein |
| CA, C48 | EhUlp1-1 | EHI_166820 | 197 | / | Ulp1 protease family |
|  | EdUlp1-1 | EDI_278410 | 197 | 87 | sentrin-specific protease |
|  | EnUlp1-1 | ENU1_195640 | 197 | 97 | Ulp1 protease family, C-terminal catalytic domain containing protein |
|  | EhUlp1-2 | EHI_097940 | 538 | / | Ulp1 protease family |
|  | EdUlp1-2 | EDI_144890 | 535 | 84 | sentrin/sumo-specific protease |
|  | EnUlp1-2 | ENU1_101400 | 536 | 96 | Ulp1 protease family, C-terminal catalytic domain containing protein |
|  | EhUlp1-3 | EHI_067510 | 285 | / | Ulp1 protease family |
|  | EdUlp1-3 | EDI_071650 | 285 | 94 | sentrin-specific protease, |
|  | EnUlp1-3 | **ENU1 –** | – | – |  |
| CA, C54 | EhAUTO1 | EHI_058500 | 325 | / | peptidase, C54 family |
|  | EdAUTO1 | EDI_281600 | 325 | 90 | hypothetical protein |
|  | EnAUTO1 | ENU1_034330 | 183^a^ | 98 | peptidase, C54 family protein |
|  | EhAUTO2 | EHI_011890 | 364 | / | peptidase, C54 family |
|  | EdAUTO2 | EDI_342370 | 359 | 96 | hypothetical protein |
|  | EnAUTO2 | ENU1_162890 | 193 | 98 | peptidase, C54 family protein |
|  | EhAUTO3 | EHI_055660 | 364 | / | peptidase, C54 family |
|  | EdAUTO3 | EDI_014170 | 346 | 97 | hypothetical protein |
|  | EnAUTO3 | ENU1_138080 | 346 | 98 | peptidase, C54 family protein |
|  | EhAUTO4 | EHI_004510 | 348 | / | peptidase, C54 family |
|  | EdAUTO4 | EDI_067040 | 348 | 95 | hypothetical protein, conserved |
|  | EnAUTO4 | ENU1_154630 | 348 | 98 | peptidase, C54 family protein |
| CA, C65 | EhOTU | EHI_064430 | 259 | / | OTU-like cysteine protease, putative |
|  | EdOTU | EDI_336440 | 259 | 90 | ubiquitin thioesterase OTU1 |
|  | EnOTU | ENU1_052870 | 262 | 97 | OTU family cysteine protease |
| **Aspartic peptidase** | |  |  |  |  |
| AD, A22, A | EhAsP22-1 | EHI_031250 | 340 | / | signal peptidase |
|  | EdAsP22-1 | EDI_129450 | 299 | 90 | minor histocompatibility antigen H13 |
|  | EnAsP22-1 | ENU1_156880 | 331 | 94 | signal peptidase |
|  | EhAsP22-2 | EHI_177160 | 316 | / | signal peptide peptidase family protein |
|  | EdAsP22-2 | EDI_192220 | 315 | 82 | minor histocompatibility antigen H13 |
|  | EnAsP22-2 | ENU1_160470 | 316 | 92 | signal peptide peptidase family protein |
|  | EhAsP22-3 | EHI_027780 | 320 | / | signal peptide peptidase family protein |
|  | EdAsP22-3 | EDI_021380 | 321 | 88 | signal peptide peptidase |
|  | EnAsP22-3 | ENU1_185560 | 321 | 98 | signal peptide peptidase family protein |
|  | EhAsP22-4 | EHI_188280 | 396 | / | presenilin 1 peptidase |
|  | EdAsP22-4 | EDI_158600 | 233 | 98 | presenilin hop-1 |
|  | EnAsP22-4 | ENU1_184090 | 396 | 99 | presenilin 1 peptidase |
| **Serine peptidase** | |  |  |  |  |
| SC, S9, C | EhSP9-2 | EHI_136440 | 665 | / | dipeptidyl-peptidase |
|  |  | EDI_096060 | 665 | 95 | dipeptidyl-peptidase 5 precursor |
|  |  | ENU1_163370 | 559 | 98 | prolyl oligopeptidase family protein |
|  | EhSP9-3 | EHI_153350 | 656 | / | prolyl oligopeptidase family protein |
|  |  | EDI_093520 | 559 | 94 | dipeptidyl-peptidase 5 precursor |
|  |  | ENU1_163370 |  | 98 | prolyl oligopeptidase family protein |
|  | EhSP9-4 | EHI_182720 | 669 | / | dipeptidyl-peptidase, |
|  |  | EDI_251670 | 669 | 98 | dipeptidyl-peptidase 5 precursor |
|  |  | ENU1_013480 | 669 | 92 | dipeptidyl-peptidase |
| SF, S26, B | EhSP26-1 | EHI_197020 | 189 | / | signal peptidase |
|  |  | EDI_121940 | 178 | 92 | signal peptidase complex catalytic subunit SEC11A |
|  |  | ENU1_022100 | 189 | 97 | signal peptidase I protein |
|  | EhSP26-2 | EHI_121860 | 168 | / | microsomal signal peptidase subunit |
|  |  | EDI_123560 | 168 | 83 | microsomal signal peptidase 23 kD subunit |
|  |  | **ENU1 –** | – | – |  |
| SC, S28 | EhSP28-1 | EHI_068090 | 480 | / | serine carboxypeptidase (S28) family protein |
|  |  | EDI_144390 | 480 | 96 | hypothetical protein |
|  |  | **ENU1_143360** | 466 | 38 | serine carboxypeptidase (S28) family protein |
|  | EhSP28-2 | EHI_037190 | 480 | / | serine carboxypeptidase (S28) family protein |
|  |  | EDI_284230 | 480 | 95 | hypothetical protein |
|  |  | **ENU1_143360** | 466 | 39 | serine carboxypeptidase (S28) family protein |
|  | EhSP28-3 | EHI_054530 | 466 | / | serine carboxypeptidase (S28) family protein |
|  |  | EDI_079160 | 466 | 94 | hypothetical protein |
|  |  | ENU1_143360 | 466 | 99 | serine carboxypeptidase (S28) family protein |
| ST, S54 | EhROM1 | EHI_197460 | 304 | / | peptidase S54 (rhomboid) family protein |
|  |  | EDI_002800 | 334 | 95 | peptidase S54 (rhomboid) family protein |
|  |  | ENU1_000930 | 330 | 99 | peptidase S54 (rhomboid) family protein |
| **Metallo peptidase** | |  |  |  |  |
| MA, M1 | EhMP1-1 | EHI_008380 | 827 | / | aminopeptidase |
|  |  | EDI_061590 | 827 | 94 | puromycin-sensitive aminopeptidase |
|  |  | ENU1_022670 | 827 | 99 | aminopeptidase |
| MA, M3 | EhMP3-1 | EHI_001080 | 675 | / | hypothetical protein |
|  |  | EDI_319300 | 675 | 90 | oligopeptidase A |
|  |  | ENU1_201170 | 700 | 96 | oligopeptidase A |
|  | EhMP3-2 | EHI_185560 | 710 | / | oligopeptidase A |
|  |  | EDI_072420 | 710 | 91 | oligopeptidase A |
|  |  | ENU1_166520 | 710 | 98 | oligopeptidase A |
| MA, M8 | EhMP8-1 | EHI_200230 | 643 | / | cell surface protease gp63 |
|  |  | **EDI_037980** | 662 | 34 | hypothetical protein |
|  |  | ENU1_098770 | 643 | 99 | cell surface protease gp63 |
|  | EhMP8-2 | EHI_042870 | 662 | / | cell surface protease gp63, |
|  |  | EDI_037980 | 662 | 92 | hypothetical protein |
|  |  | ENU1_206570 | 662 | 99 | cell surface protease gp63 |
| MA, M48, A | EhMP48-1 | EHI_075660 | 416 | / | CAAX prenyl protease |
|  |  | EDI_077050 | 416 | 93 | caax prenyl protease ste24 |
|  |  | ENU1_204680 | 416 | 97 | CAAX prenyl protease |
| ME, M16, C | EhMP16-1 | EHI_124430 | 969 | / | Zn-dependent peptidase |
|  |  | EDI_023860 | 941 | 87 | protein hypA |
|  |  | ENU1_200330 | 969 | 98 | Zn-dependent peptidase |
| MG, M24, A | EhMP24-1 | EHI_126880 | 413 | / | methionine aminopeptidase |
|  |  | EDI_335240 | 413 | 98 | methionine aminopeptidas |
|  |  | ENU1_192470 | 413 | 99 | methionine aminopeptidase, type II protein |
|  | EhMP24-2 | EHI_010070 | 471 | / | Xaa-Pro dipeptidase |
|  |  | EDI_107330 | 471 | 97 | Xaa-Pro dipeptidase |
|  |  | ENU1_214880 | 471 | 99 | Xaa-Pro dipeptidase |
|  | EhMP24-3 | EHI_140630 | 577 | / | hypothetical protein |
|  |  | EDI_204310 | 477 | 93 | xaa-pro aminopeptidase, |
|  |  | ENU1_001990 | 577 | 97 | aminopeptidase, |
|  | EhMP24-5 | EHI_115380 | 589 | / | aminopeptidase |
|  |  | EDI_258920 | 582 | 96 | xaa-pro aminopeptidase |
|  |  | ENU1_068690 | 589 | 99 | aminopeptidase |
|  | EhMP24-6 | EHI_175020 | 372 | / | peptidase |
|  |  | EDI_139430 | 371 | 96 | proliferation-associated protein 2G4 |
|  |  | ENU1_077020 | 372 | 99 | peptidase |
| MH, M18 | EhMP18-1 | EHI_021340 | 435 | / | aminopeptidase |
|  |  | EDI_150800 | 435 | 96 | hypothetical protein |
|  |  | ENU1_007410 | 435 | 99 | aminopeptidase |
|  | EhMP18-2 | EHI_106690 | 431 | / | aspartyl aminopeptidase |
|  |  | EDI_155340 | 454 | 93 | hypothetical protein |
|  |  | ENU1_200600 | 431 | 99 | aspartyl aminopeptidase |
| MH, M20, B | EhMP20-1 | EHI_186930 | 379 | / | peptidase T |
|  |  | EDI_016640 | 379 | 93 | peptidase T |
|  |  | ENU1_166190 | 379 | 99 | peptidase T |
|  | EhMP20-2 | EHI_158320 | 401 | / | peptidase T |
|  |  | EDI_216570 | 401 | 95 | peptidase T |
|  |  | ENU1_148220 | 401 | 99 | peptidase T protein |
|  | EhMP20-3 | EHI_042170 | 516 | / | aminoacyl-histidine dipeptidase |
|  |  | EDI_165910 | 516 | 97 | aminoacyl-histidine dipeptidase |
|  |  | ENU1_073770 | 516 | 99 | aminoacyl-histidine dipeptidase |
|  | EhMP20-4 | EHI_127280 | 505 | / | aminoacyl-histidine dipeptidase |
|  |  | EDI_190630 | 505 | 92 | aminoacyl-histidine dipeptidase |
|  |  | ENU1_122370 | 505 | 99 | aminoacyl-histidine dipeptidase |
| MK, M22 | EhMP22-1 | EHI_024630 | 335 | / | glycoprotein endopeptidase |
|  |  | EDI_342200 | 335 | 99 | O-sialoglycoprotein endopeptidase |
|  |  | ENU1_013970 | 335 | 99 | glycoprotein endopeptidase |
| M, M49 | EhMP49-1 | EHI_110750 | 645 | / | dipeptidyl-peptidase III |
|  |  | EDI_340680 | 645 | 95 | dipeptidyl peptidase III |
|  |  | ENU1_089520 | 645 | 99 | dipeptidyl-peptidase III |
| U, U48 | EhU48-1 | EHI_187200 | 237 | / | CAAX prenyl protease family |
|  |  | **EDI –** | – | – |  |
|  |  | ENU1_030830 | 237 | 99 | CAAX prenyl protease family protein |
